# The Impact of Orthography on Text Production in Three Languages: Catalan, English, and Spanish

**DOI:** 10.3389/fpsyg.2020.00878

**Published:** 2020-06-03

**Authors:** Anna Llaurado, Julie E. Dockrell

**Affiliations:** Institute of Education, University College London, London, United Kingdom

**Keywords:** spelling, reading, writing system, text production, cross linguistic comparison

## Abstract

Learning to write effectively is key for learning and participation in social communities. In English, transcription skills (handwriting and spelling) constrain written production at the early stages of learning to write. The effect of transcription diminishes with age, when reading skills enhance text production. Less is known about how transcription and reading interact with writing in other languages. In this study, we explore the relationships between spelling, reading and the length and quality of written text produced by primary school children speaking three different languages: Catalan, English, and Spanish. These languages are good test cases for models of writing development as they contrast orthographically and morphologically. Participants produced a written narrative text and completed standardized assessments of handwriting, spelling, reading decoding, and reading comprehension. Language had a significant effect on text production measures: young Spanish children produced longer texts which were of higher quality than the other two cohorts. They also produced the lowest number of spelling errors both at the root and for affixed morphemes. By contrast, the English children produced the highest number of both types of errors. The Catalan children did not differ significantly from their English peers for root level spelling but produced significantly fewer spelling errors at the affixed morpheme level. To test how transcription and reading skills impact on text production skills, we conducted regression analysis for each language. Different patterns of relationships between transcription, reading and text production emerged. In Catalan only handwriting fluency accounted for significant variance in text productivity and quality. By contrast, for the English children significant variance in productivity was accounted for by reading and handwriting fluency and for text quality by handwriting fluency and spelling. For the Spanish children reading skills were the significant factor for text quality. No other models were significant. Implications for developmental models of writing development are discussed.

## Introduction

Learning to write effectively and efficiently is a foundational skill for both learning at school and gaining employment in the workplace. To date models of writing development have been primarily developed from studies of children learning to write in English (Juel, 1988; Berninger et al., 2002; Berninger and Winn, 2006). In this study, we explore the relationships between spelling, reading, and written text production in children in primary schools speaking three different languages: Catalan, English, and Spanish. Our focus is on the differential performance in spelling between orthographies and, how these skills underpin written text productivity and quality across the elementary school years.

Orthographies place different demands on children’s language and literacy skills. Significant advances have been made in our understanding of the processes that underpin reading decoding and single word spelling by comparing performance across orthographies (see for example, Moll et al., 2014; Landerl et al., 2019), but much less is known about the ways in which the interplay between orthographic differences and language typologies influence writing performance and writing development. To capture differences comparisons of children writing in different orthographies using similar measures with comparable analytic approaches are needed. We address this gap in the current literature by examining pupil performance with the same measures within the same study design across three languages, which vary in orthographic consistency and morphological complexity. To our knowledge this is the first study to compare spelling and written products across three languages.

### Producing Written Text

Producing a piece of written text requires the writer to generate ideas and represent these in a symbolic form. The “simple view of writing” (Berninger et al., 1992; Berninger and Amtmann, 2003) and, later, the “not-so-simple view of writing” (Berninger and Winn, 2006) have conceptualized the multiple components of the writing system. The model synthesizes diverse trends in compositional research whereby transcription skills (handwriting or typing and spelling) and executive functions enable text generation at word sentence and text level. More recently the role of more distal factors such as oral language and reading have been incorporated into these models (see for example, Kim and Park, 2019). There is increasing evidence that two key dimensions of the writing product capture writing development in elementary school children: productivity and text quality (see for example, Berninger and Swanson, 1994; Graham et al., 1997; Olinghouse and Graham, 2009).

For novice writers, especially in English, spelling skills are thought to limit the efficiency of translation (see Kent and Wanzek, 2016, for a recent meta analysis). In English spelling requires a substantial allocation of memory resources and executive control for young writers and a lack of fluency in spelling directly constrains productivity and the quality of written texts (Graham et al., 1997; Moll et al., 2014). The demands of single word spelling, effectively, limit the cognitive resources available for the linguistic generation of the text, and thereby reduce the potential impact of other skills on the quality of early written compositions. However, children who learn to write in languages other than English may encounter different difficulties in producing written texts. For example, languages such as Italian, Turkish, and Greek have more shallow orthographies than English, but a more complex inflectional morphology (Nikolopoulos et al., 2006; Babayigit and Stainthorp, 2011; Arfe et al., 2016). For transparent orthographies the regularity of the orthography reduces the demands in generating written texts at the single word level, that is spelling, however, the complexity of the morphology of the language increases the demand on text generation (Berman, 2014; Reilly et al., 2014; Arfe et al., 2016).

While beginning writing is underpinned by spelling skills, in English reading also influences written text production. Word reading skills are associated with spelling skills (Abbott and Berninger, 1993) and word recognition skills consistently predict spelling skills at all elementary year grades (Abbott et al., 2010). Improvement in word reading leads to an improvement in spelling (Ahmed et al., 2014). Additionally, reading decoding is a good predictor of orthography consistent rule learning as reading decoding supports orthographic knowledge in spelling development (Caravolas et al., 2012). There is thus consistent evidence that single word decoding supports single word spelling. By corollary, poor reading comprehension impacts on text level writing, where pupils with lower reading comprehension, but age appropriate spelling ability, produce texts which are less sophisticated and more limited in comparison to their age matched peers (Cragg and Nation, 2006). Recent findings suggests that reading-to-writing models, that is reading supports writing, are superior, especially for the word and text levels of writing in elementary school (Ahmed et al., 2014). Yet only moderate associations between writing and measures of reading are reported for writers in English (Kent and Wanzek, 2016), perhaps reflecting the nature of the orthography. Reading skills are likely to become more important once basic spelling skills are mastered and in languages where spelling causes fewer challenges for children.

### The Impact of Orthography on Learning to Write

While there are a few exceptions, studies typically focus on spelling and written text production in English, a deep orthography where learning to spell can be challenging (but see Caravolas, 2004; Caravolas et al., 2012). Indeed, English has been described as an “outlier orthography” in terms of the inconsistency of its phoneme to grapheme correspondences and regarded as the least consistent of any alphabetic orthography (Ziegler et al., 1997). By contrast single word reading and single word spelling skills are learned more quickly in more consistent orthographies (Landerl et al., 1997). Studies of reading acquisition commonly demonstrate that rates differ between children learning opaque and transparent orthographies. In English, the rate of development is twice as slow for reading as in more shallow orthographies (Seymour et al., 2003). Similarly, Wimmer and Landerl (1997) observed faster single word spelling development rates in German, considered a more transparent orthography than English. In a recent comparison of the spelling accuracy of English and Italian speaking pupils in grades 2–5, Marinelli et al. (2015) demonstrated both faster rates of spelling development in Italian and more persistent cross-linguistic gaps in spelling than in reading accuracy, suggesting that spelling accuracy, but not reading accuracy, is moderated by orthographic consistency. Furthermore, the inconsistency of orthographies is usually stronger from phoneme to grapheme than from grapheme to phoneme; making spelling a particularly challenging skill to master in inconsistent orthographies (see Ziegler et al., 1996, 1997). However, these studies have not considered the relationships between orthography and written text production. In this study, we examine the extent to which the orthographies of three languages (Catalan, English, and Spanish) that contrast in their orthographic consistency underpin spelling development and written text production.

## Language Typologies

Spanish, Catalan, and English reflect the continuum of orthographies where Spanish, a highly transparent orthography has consistent relationships from phoneme to grapheme and grapheme to phoneme (nearly 100% of the letters have one phoneme only and nearly 90% of phonemes are represented by only one grapheme). By contrast, English is characterized by a high level of inconsistency in both reading and spelling (only 72% of the letters have a single phoneme and 62% of the phonemes can be represented by only one grapheme). Catalan contrasts with both Spanish and English. It is neither as transparent as the Spanish orthography nor as opaque as the English orthography [where 76% of the letters represent only one phoneme 70% of the phonemes can be represented by only one grapheme (ERN-LWE) COST-Action IS0703 Spelling Report; Caravolas et al., 2012]. The Catalan and Spanish orthographic systems also differ from English in their use of a graphic accent to mark the stressed syllable in a number of words. In Catalan the phonological content of the vowel requires different graphic marks, in cases where one vowel represents more than one phoneme. In addition to drawing on phoneme grapheme conversion processes spelling in Catalan and Spanish requires knowledge and use of the prosody of the word. The conventional use of the accent system is usually acquired after children have mastered the phoneme to letter correspondences (Defior et al., 2009).

The three languages examined in this study also contrast in their morphological systems. English, a Germanic language, has a sparse morphology, particularly at the inflectional level whereas Catalan and Spanish, both Romance languages, have rich morphologies. English typically uses three to four morphemes to encode person, tense and aspect. For instance, the s morpheme of the present tense suffices to differentiate the third singular person. In Spanish and Catalan up to 47 different morphemes are used to inflect determinants, nouns and adjectives for number and gender, and verbs for aspect, mode, tense, person, and number, in contrast with the previous example for English, both Spanish and Catalan use 18 different suffixes to mark each person of the present tense paradigm (Alarcos, 2007). The accurate use of these morphological markers underpins text quality.

Morphological information is particularly important in the spelling of low frequency words (Defior et al., 2008), a potential indicator of higher quality texts (Olinghouse and Graham, 2009). At least with alphabetic orthographies, the use of morphological cues in children’s spelling would appear to differ depending on the interplay between the characteristics of the orthographic system and the morphological structure of the language. Ravid and Gillis (2006) showed that young children speaking Hebrew, a language with a highly synthetic morphology, used morphological cues to a greater extent than children of the same age who speak Dutch, a language with a much sparser morphology. A similar effect of a salient morphology was shown for children learning to spell in Spanish (Defior et al., 2009) and Catalan (Llaurado and Tolchinsky, 2016). These studies used a single word spelling task, so whether children use morphological cues to support their text based spelling and improve text quality needs elucidation.

These differences between the three languages studied are predicted to affect both the children’s spelling and also their written texts. As has been demonstrated previously we predicted that the Spanish transparent orthography will lead to few (if any spelling errors) whereas for Catalan and English spelling will be compromised in the early stages of learning to write. However, we anticipated that the more prominent morphology of Catalan would reduce the presence of affixed morpheme based errors in this language relative to English. The previously under researched role of accents is predicted to affect the children’s spelling and writing in different ways. In both Catalan and Spanish we expected missing accents to be an important cause source of spelling error. Moreover we anticipated that this will have a greater influence on the spelling of children in Catalan because spelling words conventionally is more challenging in Catalan than in Spanish and in Catalan children must learn where to use an accent and which kind of accent they must use.

## Assessing Written Text

The assessment of children’s writing raises challenges both conceptually and methodologically (see Dockrell et al., 2019). Both holistic scoring and analytic scoring of writing products have been used to capture writing development (Abbott and Berninger, 1993; Scott and Windsor, 2000; Mackie and Dockrell, 2004; McMaster and Espin, 2007; Lee et al., 2010; Wagner et al., 2011; Puranik and AlOtaiba, 2012). Analytic scoring provides a more detailed and comprehensive scoring system. One approach distinguishes the macrostructure and the microstructure of the texts produced, capturing the two key dimensions identified of productivity and text quality (Berninger and Swanson, 1994; Graham et al., 1997; Olinghouse and Graham, 2009).

Analyses of texts at the macrostructure level typically focuses on the use of a bespoke holistic scoring scale (see for example, Koutsoftas and Gray, 2012) which captures quality. Productivity by contrast is typically a text level microstructure measure of length in either total number of words or sentences produced. Research has also considered microstructure in more detail including, for example, both the nature of students’ spelling errors, lexical diversity or grammatical accuracy.

How differences in the writing systems influence the ways in which language and literacy underpin the development of the written product has begun to be explored within cohorts speaking different languages. In English, it is well established that spelling skills underpin both text productivity and text quality (Graham et al., 1997; Berninger et al., 2006; Kim et al., 2015). Different patterns have been observed in other languages. Babayigit and Stainthorp (2011) studying children writing in Turkish, a relatively transparent orthography, found that transcription skills and reading comprehension were related to text quality rather than productivity rates. Similarly, in Italian, another transparent orthography, the spelling skills of the Italian children explained text quality but not text productivity (Arfe et al., 2016). These differences between English and more transparent orthographies may reflect differences in developmental processes but may equally be explained by the use of different measures or different analytic techniques. To further our understanding of how the relationship between spelling skills and written text production relate, studies mapping equivalent processes, using comparable measures at similar developmental phases are needed.

There is clear evidence to suggest that there will be differences across the orthographies in children’s spelling competence. However, the impact of these spelling differences on the written products and the nature of the errors produced is less clear. Spelling errors reflect particular aspects of the language that challenge children. Catalan, English, and Spanish are likely to pose different spelling challenges due to their orthographic differences. These differences can be captured by analyzing the types of written errors that are produced. A number of systems exist to capture the nature of children’s spelling errors and these microstructure analyses should reflect the languages studied. Error analysis of children’s spelling at phonological, orthographic, and morphological levels can highlight the differential impact of these processes on text production (Share, 2008; Critten et al., 2014). Moreover, morphological analysis of both base words and bound morphemes (see Nagy et al., 2006) provides detailed information about the developing writer’s skills (Nunes et al., 1997). In sum, bound morphemes such as inflectional and derivational morphemes play an important role when constructing meaningful text and may represent and increased source of difficulty for text generation in languages with complex morphological systems.

## The Current Study

To examine these questions, and as part of a larger study, three different age groups of Catalan, English, and Spanish children produced a narrative writing task and completed standardized assessments of handwriting, spelling, reading decoding, and reading comprehension. We predicted that the transparency of Spanish would result in few spelling errors, increased productivity and as a result higher quality texts. Given the children’s spelling performance we anticipated a reduced impact of spelling on text productivity and text quality. However, given the role of reading on the written text of good spellers in transparent orthographies we anticipated that text quality would be predicted by the children’s reading levels. For English children we anticipated that they would be least productive and produce the lowest quality texts, especially at the younger ages, given the challenges they face with spelling. As has been found in previous studies in English, we predicted that both spelling and handwriting would underpin written productivity and text quality. There have been few studies exploring Catalan but we anticipated that the children would produce more spelling errors that the Spanish children but significantly fewer than the English children, particularly with affixes given the more prominent morphology in the language. For children writing in Catalan we predicted writing productivity to be underpinned by spelling and handwriting skills but given the morphological complexity of the language that writing quality would be underpinned by transcription and reading.

## Methods

### Participants

Two hundred and eighty-four elementary school pupils from England (*n* = 86), Catalonia (*n* = 113), and Spain (*n* = 85) participated in this study. Pupils attended year 2, 4, and 6 in three schools, one in each region, which were purposely selected to reflect the mainstream population. The English cohort attended a school in South East London. As Catalonia is a region with two official languages, Catalan and Spanish, the Catalan cohort was bilingual. Catalan is the only language of instruction in schools and it was the dominant language (spoken at home too) for a vast majority of the participants. The Spanish cohort attended a school in Ciudad Real, a Spanish monolingual region in Spain. All children in each year group participated in the study. No child was reported to have a hearing or visual impairment. For the English cohort, mean age in months was *M* = 87, SD = 3.96 (range 81–92) for the 31 children (15 boys) in Year 2, *M* = 111, SD = 5.63 (range 105–117) for the 27 children (11 boys) in Year 4, and *M* = 135, SD = 3.48 (range 129–140) for the 28 children (18 boys) in Year 6. For the Catalan cohort, mean age in months was *M* = 94, SD = 3.19 (range 88–99) for the 38 children (22 boys) in Year 2, *M* = 117, SD = 4.65 (range 113–123) for the 35 children (16 boys) in Year 4, and *M* = 140, SD = 3.88 (range 135–146) for the 40 children (22 boys) in Year 6. For the Spanish cohort, mean age in months was *M* = 92, SD = 3.34 (75–86) for the 26 children (13 boys) in Year 2, *M* = 116, SD = 2.97 (range 113–124) for the 29 children (16 boys) in Year 4, and *M* = 140, SD = 3.51 (range 135–146) for the 30 children (22 boys) in Year 6. The difference between the mean age of the Catalan and Spanish, and the English participants is explained by different school entry dates (England September to August, in Catalonia and Spain January to December) and age in months is controlled for in relevant analysis.

### Measures

Children were assessed on a range of measures to examine their transcription and literacy skills. All children were assessed in their first language using measures appropriate for the population.

#### Measures of Transcription

##### Handwriting fluency

Children are asked to write as many alphabet letters as possible in 1 min (Wagner et al., 2011). Children are asked to write all the alphabet letters in order, using lower case letters. If children finish writing all letters before a minute, they are asked to continue to write starting with “a” again. This task assesses how well children access, retrieve, and write alphabet letter forms automatically. All teachers confirmed that the children in their classes knew how to write the alphabet.

##### Dictated spelling

English: British Abilities Scales II (BAS II); Spelling Scale: This scale provides a number of phonetically regular and irregular words to assess the child’s ability to produce correct spellings. Each item is first presented in isolation, then within the context of a sentence, and finally in isolation. The child has to respond by writing the word and for this study 40 words were dictated to children in each year: reliability 0.91; validity with Wechsler Objective Reading Dimension (WORD) spelling 0.63.

Catalan: We used a bespoke task created by Tolchinsky (in press). Participants had to write down the words dictated by the experimenter. Each word was repeated twice before proceeding to the next one. Due to the lack of an updated Catalan word frequency dictionary the target words were selected from the Corpus Cesca; a corpus of written Catalan produced by school children (Llaurado et al., 2012) demonstrating the validity of the task. The selected words were from the same semantic field – food and the same grammatical category – nouns, and they were controlled for frequency and orthographic difficulty. Each participant had to spell a total of 40 words; four sets of words divided for frequency (high and low) and orthographic difficulty (high and low).

Spanish: The same task that was used in Catalan but adapted for Spanish (Llaurado and Tolchinsky, 2016).

#### Reading

##### Word level reading

English: Test of Word Reading Efficiency (TOWRE; Torgesen et al., 1999): This contains two subtests. The Sight Word Efficiency (SWE) subtest assesses the number of real printed words that can be accurately identified within 45 s, and the Phonetic Decoding Efficiency (PDE) subtest measures the number of pronounceable printed non-words that can be accurately decoded within 45 s.

Catalan: We adapted the PROLEC-R Lexical Processes, word and pseudoword reading for Spanish: reliability 0.79. This contains two subtests. The word reading subtest assesses the time that takes a child to accurately read a set of 40 real printed words, and the non-word reading subtest that measures the time it takes a child to accurately decode a list of 40 pronounceable printed non-words.

Spanish: We used the PROLEC-R Lexical Processes, word and pseudoword reading for Spanish: reliability 0.79. This contains two subtests. The word reading subtest assesses the time that takes a child to accurately read a set of 40 real printed words, and the nonword reading subtest that measures the time it takes a child to accurately decode a list of 40 pronounceable printed non-words.

##### Reading comprehension

English: The New Group Reading Test. This is a standardized assessment using a multiple-choice format to assess children’s ability to complete sentences and comprehend written passages. It can be administered to groups. Reliability (Cronbach’s alpha: 0.90) is high.

Catalan: ACL (Avaluació de la Comprensió Lectora). This test comprises a set of seven texts for each school year. For each text, children are requested to read it individually and then answer a set of multiple choice questions. Avaluació de la Comprensió Lectora has been extendedly used in studies on Catalan reading. It has a reliability of KR-20: 0.080–0.083. Its validity, assessed as the correlation between the results obtained by a child on ACL and the child’s teacher assessment of his/her reading comprehension skills, is of 0.99.

Spanish: ACL (Evaluación de la comprensión lectora). This test comprises a set of seven texts for each school year. For each text, children are requested to read it individually and then answer a set of multiple choice questions. Avaluació de la Comprensió Lectora has been extendedly used in studies on Spanish reading. It has a reliability of KR-20: 0.078 to 0.083. Its validity, assessed as the correlation between the results obtained by a child on ACL and the child’s teacher assessment of his/her reading comprehension skills, is of 0.97.

### Writing Measures

All children were asked to produce a written response to the prompt “What is your ideal place for a holiday like and why.” This task is based on the standardized assessment of writing in the Weschler Objective Language Dimensions test (WOLD; Weschler, 2005) and has been used in a number of studies.

### Procedure

Pupils were assessed twice, as a class group for the writing measure and individually in schools for the transcription and literacy measures. The individual assessment lasted over 50 min. The writing prompt used in the analyses was presented to the class on the second session.

To ensure all pupils were familiar with the writing activity, they were provided with an opportunity to practice the writing task with a different narrative prompt a few days before the writing assessment. These data were not included in the analyses. On the writing assessment day, pupils were asked to produce a written response to the prompt “What is your ideal holiday place like and why.” The task was not time limited, the researcher had a 50 min long class period to explain to the pupils the purpose of the task, hand out the necessary materials and deliver the task prompts. On average, pupils wrote for 20 min and no child requested extra time to finish his or her text once the time the session was over. For the three cohorts, language teachers were present in the classroom during the task. Ethical approval was secured from the authors institution (UCL-IOE for review). Informed consent from schools and parents was provided prior to any testing.

#### Transcription and Coding of Texts

##### Transcription of texts

A literal copy of all written outlines and texts was transcribed and entered in a standard format using the Systematic Analysis of Language Transcript conventions (SALT; Miller and Chapman, 2000). Systematic Analysis of Language Transcript conventions allows for the automatic coding of certain text features and for the creation of codes specifically created for the purpose of the study.

## Coding of Written Texts

All coding was done by the first author. Written texts were coded for productivity and their overall quality. Productivity was computed as the total number of words in each text, a measure that has been widely used as an indicator of compositional length (Deacon and Kirby, 2004; Kim et al., 2011). Words used in the title, when there was one were included in the total. When a child made a word segmentation mistake, we counted the number of intended words. Any deleted or crossed over words were not included in the final total. Quality was scored using a holistically scale derived from the WOLD. We present this scale in Box 1.

**Box 1. Rubric used for coding the quality of the written texts.**

0: Unintelligible text or too few words to judge the content of the text or text which was irrelevant to the target prompt.
1: Response which included a list of elements or characteristics but did not indicate why this reflected “why or how this should make an ideal place for a holiday”.
2: Included information and indicated why or how this relates to an ideal holiday place. Could either be an extensive list with no elaboration or single element or characteristic with some descriptive details about that element or characteristic.
3: Ideas (elements or characteristics) are related to each other or to the main idea provides additional descriptive information or detail.
4: Generally well written engaging the reader with ideas clearly related to each other with the addition of clarifying descriptive detail.
5: Presents a substantial amount of description and varied detail of the topic. The ideas and details are clarified with several descriptions or thorough elaboration.
6: Well written and presents clear, organized and developed descriptions of the topic. The ideas and details are clarified and related through the use of effective transitions, resulting in an overall sense of the subject. Effectiveness is enhanced through the use of vivid imagery.

## Coding of the Spelling Errors

### Word Level Spelling Errors

We calculated the number of words that were misspelt, for instance, “amagin” [amazing] was coded as one misspelt word, and divided it by the total number of words written. If the text contained words written in a language other than English, Catalan, or Spanish, respectively, we did not include these words in the final count of either the total number of misspelled words or total number of written words. This provided a score reflecting the proportion of words spelled incorrectly.

### Number of Spelling Errors

It was possible for words to contain several spelling errors. Thus the total number of spelling errors were computed, for instance “amagin” [amazing] was coded for two misspellings: A wrong letter “ama**g** in” and a missing letter “amagin[**g**],” and divided it by the total number of words (excluding those not written in the target language). This resulted in a measure of spelling errors that could be classified by type into three different error types:

#### Spelling Errors at the Root Level

We calculated the number of spelling errors in each word at the root level and divided the total number of these spelling errors by the total number of words in the text (excluding the words written in a language that was not the target language).

#### Spelling Errors at the Affix Level

We calculated the number of spelling errors in each morphologically complex word at the affixed morphemes and divided the total number of these by the total number of affixed words in the text (excluding the words written in a language that was not the target language).

#### Misspellings Due to Absence of an Accent Mark

We calculated the number of misspelling due to a missing accent mark and divided it by the total number of words that require and accent according to the orthographic norm.

## Results

A series of factorial ANOVAs were used to compare the main effects of language and year group and any interaction effect between the two on the writing measures. When interaction effects were significant subsequent ANOVAs were computed. Age was included as a covariate given the differences between countries in the start date of the school year. Zero order correlations explored the relationships between spelling, handwriting, reading, text productivity, and text quality. For each language we examined whether age, transcription skills (handwriting or spelling), or reading predicted productivity, accuracy, and quality of writing using linear regression.

### Text Productivity

**FIGURE 1:**
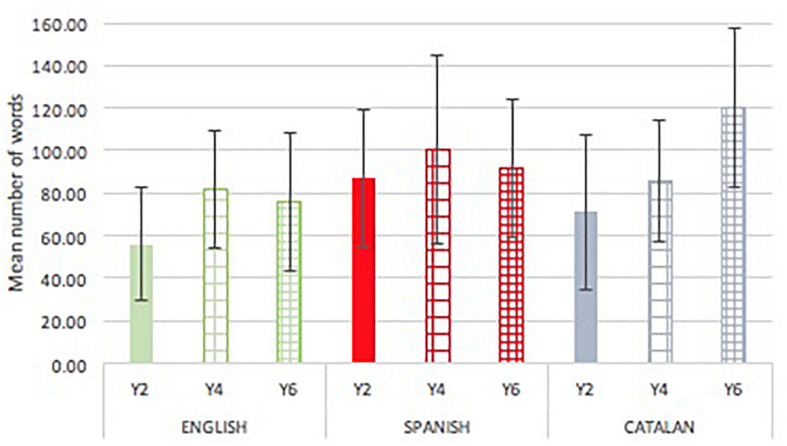
Mean (SD) text productivity (number of words) by language and school grade.

Figure 1 presents mean (SDs) of text productivity by year group and language cohort. The number of words in the written text varied significantly by language [*F*(2,284) = 12.036, *p* < 0.001, *p*η^2^ = 0.080] but not with school year [*F*(2,284) = 1.456, *p* = 0.235, *p*η^2^ = 0.011]. The interaction between school year and language was significant [*F*(4,284) = 5.676, *p* < 0.001, *p*η^2^ = 0.077]. Comparisons by language in separate analyses revealed that, in Year 2, the Spanish cohort was significantly more productive [*F*(2,94) = 6.468, *p* = 0.002, *p*η^2^ = 0.12] than their English peers (*p* = 0.001), but not more than the Catalan children (*p* = 0.158). The Catalan and English cohorts did not differ at this point (*p* = 0.178). No differences by language cohort were evident in Year 4 [*F*(2,89) = 2.484, *p* = 0.089, *p*η^2^ = 0.05]. In Year 6, language had a significant effect [*F*(2,98) = 14.323, *p* < 0.001, *p*η^2^ = 0.23] and the Catalan pupils were significantly more productive than both English (*p* = 0.001) and Spanish (*p* = 0.003) pupils, but these two did not differ significantly (*p* = 0.244). For each language, performance across year groups was examined. In English [*F*(2,86) = 6.449, *p* = 0.002, *p*η^2^ = 0.134], students in Year 2 produced significantly fewer words than students in Year 4 in English (*p* = 0.003), and in Catalan [*F*(2,113) = 20.381, *p* < 0.001, *p*η^2^ = 0.27] students in Year 4 produced significantly fewer words than in Year 6 (*p* < 0.001). In Spanish [*F*(2,85) = 0.928, *p* = 0.400, *p*η^2^ = 0.022], there were no significant changes by year group.

### Text Quality

**FIGURE 2:**
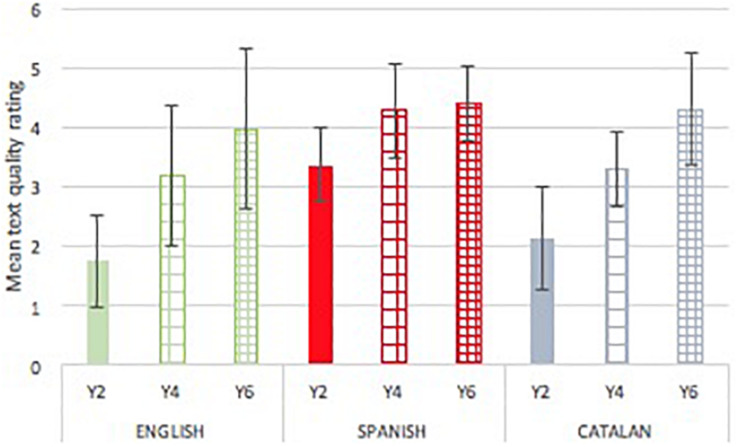
Mean (SD) text quality by language and school grade.

Figure 2 presents mean (SDs) of text quality by year group and language cohort. The quality of texts varied by language [*F*(2,284) = 38.828, *p* < 0.001, *p*η^2^ = 0.188] with a much larger language effect for text quality than for productivity, and increased with school year [*F*(2,284) = 100.449, *p* < 0.001, *p*η^2^ = 0.044]. The school year by language interaction was also significant [*F*(4,284) = 4.557, *p* = 0.001, *p*η^2^ = 0.062]. Comparisons between languages in separate analysis showed that children writing in Spanish produced significantly better quality texts that both their English and Catalan peers in Year 2 [*F*(2,94) = 32.357, *p* < 0.001, *p*η^2^ = 0.42], and Year 4 [*F*(2,92) = 14.274, *p* < 0.001, *p*η^2^ = 0.24] (*p* < 0.001 for all post-hoc contrast) but not in Year 6 [*F*(2,95) = 1.526, *p* = 0.223, *p*η^2^ = 0.03]. The differences between the English and Catalan children were not significant at any point. Children obtained higher scores for quality by year group in all the languages. For each language, performance across year groups was examined. Results revealed that, in Catalan [*F*(2,113) = 67.565, *p* < 0.001, *p*η^2^ = 0.551], English [*F*(2,86) = 30.410, *p* < 0.001, *p*η^2^ = 0.423] and Spanish [*F*(2,85) = 19.000, *p* < 0.001, *p*η^2^ = 0.317], children in Year 4 produced significantly better texts than children in Year 2 (*p* < 0.001, for all post-hoc contrasts). The contrast between Years 4 and 6 was significant in English (*p* = 0.034) and Catalan (*p* < 0.001) but not for Spanish (*p* = 1.000).

### Spelling Performance in Dictation

**FIGURE 3:**
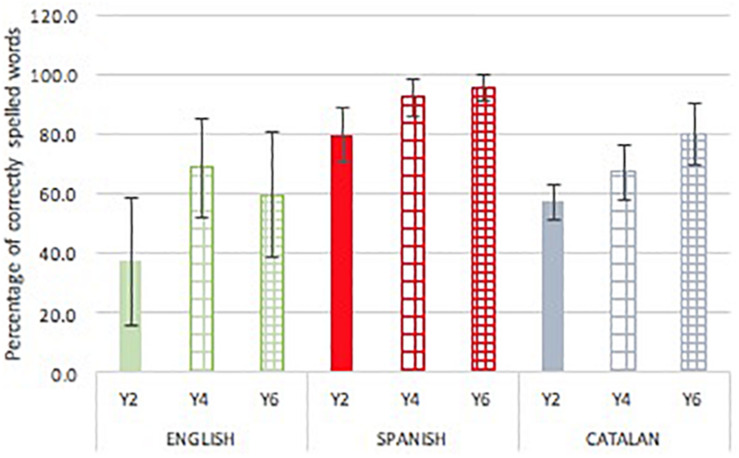
Mean (SD) percentage of correctly spelled words by language and school grade.

Both language [*F*(2,284) = 153.249, *p* < 0.001, *p*η^2^ = 0.54] and school year [*F*(2,284) = 69.409, *p* < 0.001, *p*η^2^ = 0.34] had a significant effect on performance on the single word dictation task (see Figure 3). The school year by language interaction was also significant [*F*(4,284) = 8.252, *p* = 0.001, *p*η^2^ = 0.11]. Contrasts by language in separate analysis showed significant differences between all languages in Year 2 [*F*(2,94) = 66.549, *p* < 0.001, *p*η^2^ = 0.59] and Year 6 [*F*(2,98) = 54.533, *p* < 0.001, *p*η^2^ = 0.53) (*p* < 0.001 for all post-hoc contrasts]. In Year 4 [*F*(2,92) = 46.723, *p* < 0.001, *p*η^2^ = 0.51] the difference was significant only between Spanish on the one hand, and English and Catalan on the other hand (*p* < 0.001 for both post-hoc contrasts). For each language, performance across year groups was examined. This showed that performance increased in English [*F*(2,86) = 19.129, *p* < 0.001, *p*η^2^ = 0.32], Catalan [*F*(2,113) = 67.698, *p* < 0.001, *p*η^2^ = 0.55], and Spanish [*F*(2,85) = 39.904, *p* < 0.001, *p*η^2^ = 0.49], between Years 2 and 4 (*p* < 0.001 for all post-hoc contrasts), with the Year 4 pupils spelling more words correctly. The Year 4 and Year 6 contrast, however, was only significant for the Catalan children (*p* < 0.001).

### Spelling Performance in Narrative Writing

**FIGURE 4:**
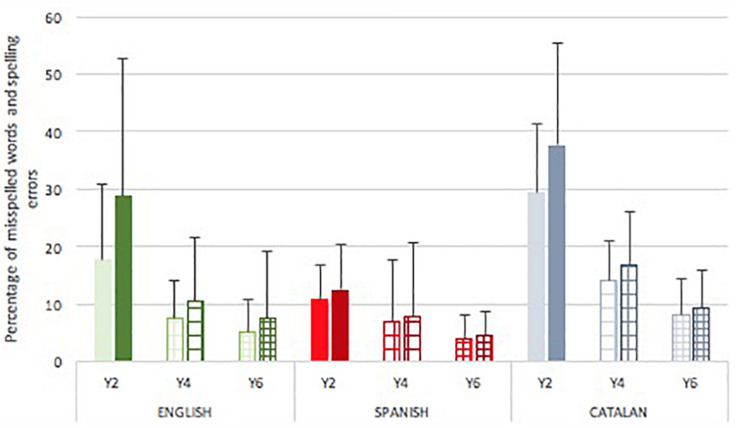
Mean (SD) percentage of misspelled words (left column) and spelling errors (right column) by language and school grade.

The numbers of words with spelling errors were examined. The main effect of language [*F*(2,283) = 35.256, *p* < 0.001, *p*η^2^ = 0.20] and school year [*F*(2,283) = 60.912, *p* < 0.001, *p*η^2^ = 0.31] were significant for the proportion of spelling errors students produced in their written texts. The school year by language interaction was also significant [*F*(2,283) = 5.911, *p* < 0.001, *p*η^2^ = 0.08]. Figure 4 shows that the Catalan cohort produced the highest proportion of words with spelling errors at all school years and Spanish cohort the lowest. Comparisons by language in separate analysis revealed that, in Year 2 [*F*(2,93) = 22.726, *p* < 0.001, *p*η^2^ = 0.34], the Catalan cohort produced a significantly higher proportion of misspelled words compared to English and Spanish (*p* < 0.001 for both post-hoc contrasts), and this repeated in Year 4 [*F*(2,92) = 7.948, *p* = 0.001, *p*η^2^ = 0.15] (*p* = 006, *p* = 002 for the two post-hoc contrasts, respectively). In Year 6 [*F*(2,98) = 5.675, *p* = 0.005, *p*η^2^ = 0.11], Catalan children still misspelled a higher proportion of words than any of the two other cohorts but only the differences between Catalan and Spanish remained significant (*p* = 006). Comparisons by school year in separate analysis showed that the English children [*F*(2,86) = 15.692, *p* < 0.001, *p*η^2^ = 0.27] misspelled a significantly lower number of words in Year 4 than in Year 2 (*p* < 0.001), but the contrast between Years 6 and 4 was not significant. The Spanish cohort [*F*(2,84) = 5.816, *p* = 0.004, *p*η^2^ = 0.13] showed only one significant contrast between Years 6 and 2 (*p* = 003). Finally, the Catalan children [*F*(2,113) = 62.193, *p* < 0.001, *p*η^2^ = 0.53] showed a steady improvement with significant contrasts between both Year 4 and Year 2 (*p* < 0.001), and Year 6 and Year 4 (*p* = 008).

### Numbers of Spelling Errors in Narrative Texts

The numbers of spelling errors in the text were examined. There were main effects of language [*F*(2,281) 22.876, *p* < 0.001, *p*η^2^ = 0.14] and school year [*F*(2,283) = 53.331, *p* < 0.001, *p*η^2^ = 0.28] and a school year by language interaction [*F*(2,281) = 5.180, *p* < 0.001, *p*η^2^ = 0.07]. Figure 4 shows that the Catalan cohort produced the highest proportion of spelling errors at all school years and Spanish the lowest. Comparisons by language in separate analysis revealed that, in Year 2 [*F*(2,93) = 14.370, *p* < 0.001, *p*η^2^ = 0.24], the Spanish cohort produced a significantly smaller proportion of spelling errors than their English (*p* = 0.004) and Catalan (*p* < 0.001) peers, and that the language effect remained significant over time in Year 4 [*F*(2,92) = 5.632, *p* = 0.005, *p*η^2^ = 0.11] and, marginally, Year 6 [*F*(2,98) = 3.226, *p* = 0.042, *p*η^2^ = 0.06] between Spanish and Catalan (*p* = 0.005, *p* = 0.038, respectively). Comparisons by year group in separate analysis showed the same pattern as in number of misspelled words. Thus, the English children made a significantly lower number of misspelling [*F*(2,86) = 13.846, *p* < 0.001, *p*η^2^ = 0.25] in Year 4 than in Year 2, *p* < 0.001, but the contrast between Years 6 and 4 was not significant. The Spanish cohort showed only one significant contrast [*F*(2,84) = 5.510, *p* = 0.006, *p*η^2^ = 0.12] between Years 5 and 1 (*p* = 0.004). With age, the Catalan children made significantly fewer spelling errors [*F*(2,113) = 56.588, *p* < 0.001, *p*η^2^ = 0.51] and contrasts were significant between both Year 4 and Year 2 (*p* < 0.001), and Year 6 and Year 4 (*p* = 023).

### Types of Spelling Errors in Narrative Texts

**FIGURE 5:**
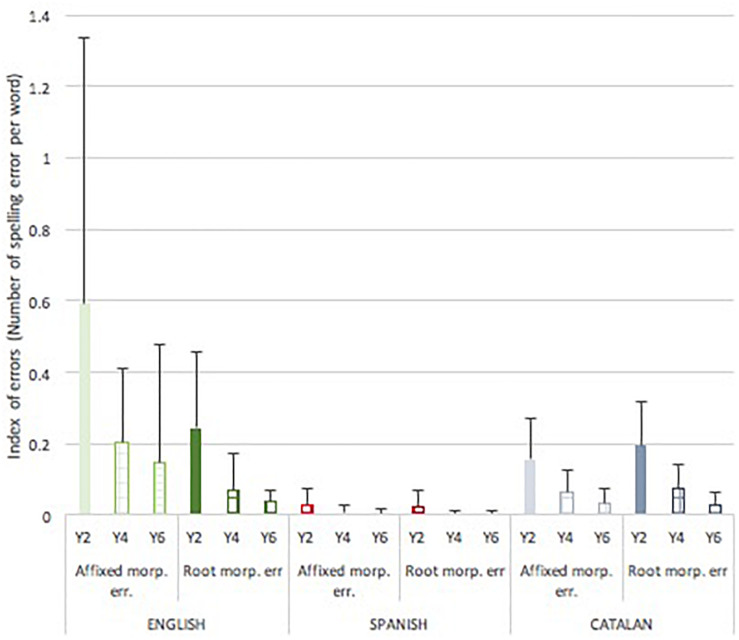
Mean (SD) index of spelling number of spelling errors at the root level per word and at the morpheme level per morphologically complex word by language and school grade.

The proportions of spelling errors at the root and affix level were examined (Figure 5). For root level errors there was a significant effect of language [*F*(2,282) = 24.41, *p* < 0.001, *p*η^2^ = 0.15] and year group [*F*(2,282) = 42.56, *p* < 0.001, *p*η^2^ = 0.24] and a significant interaction between language and year group [*F*(4,282) = 24.41, *p* < 0.001, *p*η^2^ = 0.09]. The children writing in Spanish made significantly fewer spelling errors than both their English, *p* < 0.001 and Catalan, *p* < 0.001 peers. Comparisons by language in separate analyses showed that, in year 2 [*F*(2,93) = 14.971, *p* < 0.001, *p*η^2^ = 0.25], the pupils writing in Spanish made significantly fewer root level errors than pupils writing in English (*p* < 0.001), or Catalan (*p* < 0.001); in year 4, the difference remained significant [*F*(2,92) = 4.533, *p* = 0.013, *p*η^2^ = 0.09] between the Spanish and the Catalan children (*p* = 0.018) but was only nearly significant between the Spanish and the English children (*p* = 0.0.66); finally in year 6, only the Spanish and the English children showed significant differences [*F*(2,98) = 6.181, *p* = 0.003, *p*η^2^ = 0.12] (*p* = 0.002 for the post-hoc contrast). Age had a different effect depending on the language; the number of root errors decreased significantly in English [*F*(2,86) = 14.648, *p* < 0.001, *p*η^2^ = 0.26] and Catalan [*F*(2,113) = 41.439, *p* < 0.001, *p*η^2^ = 0.43] between Years 2 and 4 only (*p* < 0.001) (though in Catalan the decrease between Years 4 and 6 almost reached significance *p* = 0.050); whereas in Spanish [*F*(2,84) = 6.260, *p* = 0.003, *p*η^2^ = 0.13] the decrease occurred at a slower pace and was significant between Years 2 and 6 (*p* = 0.002).

For misspelled affixes there was also significant effect of language [*F*(2,279) = 28.44, *p* < 0.001, *p*η^2^ = 0.17] and year group [*F*(2,279) = 13.92, *p* < 0.001, *p*η^2^ = 0.09] and a significant interaction between language and year group [*F*(4,279) = 5.63, *p* < 0.001, *p*η^2^ = 0.08]. Comparisons by language in separate analyses showed that the children writing in English made more affix errors in Year 2 [*F*(2,91) = 13.817, *p* < 0.001, *p*η^2^ = 0.24] and Year 4 [*F*(2,92) = 19.655, *p* < 0.001, *p*η^2^ = 0.31] than the children writing in Spanish and Catalan (all, *p* < 0.001), and again in Year 6 [*F*(2,97) = 5.208, *p* = 0.007, *p*η^2^ = 0.10] (*p* = 0.002 and *p* = 0.032 for post-hoc contrasts with Spanish and Catalan, respectively). Children writing in Catalan or Spanish did not differ at any age point regarding their ability to spell word affixes. For each language, performance across year groups was examined. Children significantly improved the spelling of affixes in English [*F*(2,83) = 6.976, *p* = 0.002, *p*η^2^ = 0.15] with significant post-hoc contrast between Years 2 and 4 (*p* = 0.012), in Catalan [*F*(2,113) = 26.827, *p* < 0.001, *p*η^2^ = 0.33] also with a significant post-hoc contrast between Years 2 and 4 (*p* < 0.001), and Spanish [*F*(2,84) = 4.102, *p* = 0.020, *p*η^2^ = 0.09] with a significant post-hoc contrast between Years 2 and 6 (*p* = 0.027).

Omission of accents in Catalan and Spanish was compared. There was a significant effect of language [*F*(1,189) = 4.34, *p* = 0.04, *p*η^2^ = 0.02] and year group [*F*(1,189) = 29.97, *p* < 0.001, *p*η^2^ = 0.25] but no interaction [*F*(2,189) = 0.09 *ns*.]. Children writing in Catalan omitted more accents in comparison to students writing in Spanish, *p* < 0.001, as did children in the lower year groups (Year 2 in contrast to Year 4, *p* < 0.001 and Year 6, *p* < 0.001).

### The Impact of Orthography on the Relationships Between Transcription, Reading and Writing

We first explored the zero order correlations between the writing measures of numbers of words produced and text quality with transcription (handwriting and dictated word spelling) and reading (word reading and reading comprehension) for each language cohort (see Table 1 for *M*(SD) and Table 2 for correlations). Bonferroni’s adjustment (*p* = 0.008 for significance) was used to control for multiple correlations. As expected, in English both measures of transcription and reading comprehension were significantly associated with text productivity and text quality. For Catalan a similar pattern was evident but reading comprehension was not significantly associated with text productivity. There was a more mixed pattern for Spanish where written text productivity was only significantly associated with reading comprehension but text quality was associated with both reading and transcription skills.

**TABLE 1:** Descriptive *M*(SD) for transcription and reading skills.

	Year 2			Year 4			Year 6		
			
	Eng	Cat	Spa	Eng	Cat	Spa	Eng	Cat	Spa
	(*N* = 31)	(*N* = 38)	(*N* = 26)	(*N* = 27)	(*N* = 35)	(*N* = 29)	(*N* = 28)	(*N* = 40)	(*N* = 30)
Hand writing fluency	26.42 (14.01)	17.03 (8.57)	25.15 (9.49)	40.15 (18.10)	43.63 (19.59)	34.55 (14.69)	47.04 (20.50)	63.15 (16.56)	54.13 (19.68)
Dictated word spelling	20.82 (9.76)	18.23 (1.91)	25.48 (2.99)	2735 (6.60)	21.46 (3.00)	29.43 (2.05)	23.71 (8.40)	25.52 (3.30)	30.59 (1.43)
Word reading	45.3 (15.70)	44.26 (18.55)	85.92 (34.31)	65.93 (11.01)	96.58 (42.19)	133.47 (32.31)	70.68 (11.73)	136.76 (40.51)	173.71 (31.01)
Reading comprehension	26.13 (11.94)	17.84 (2.96)	18.08 (4.14)	42.74 (4.84)	19.29 (5.23)	17.62 (4.59)	30.96 (10.10)	19.62 (5.46)	20.20 (3.02)

**TABLE 2:** Zero order correlations between transcription, reading, and writing measures of productivity and quality by language.

Language		Handwriting fluency	Single word spelling	Word reading	Reading comprehension	Text productivity
**English**						
*N* = 86	Single word spelling	0.48**				
	Word reading	0.60**	0.72**			
	Reading comprehension	0.35**	0.71**	0.66**		
	Text productivity	0.44**	0.36**	0.42**	0.47**	
	Text quality	0.58**	0.49**	0.68**	0.43**	0.66**
**Spanish**						
*N* = 85	Single word spelling	0.50**				
	Word reading	0.67**	0.77**			
	Reading comprehension	0.21	0.38**	0.38**		
	Text productivity	0.11	0.12	0.12	0.24*	
	Text quality	0.32*	0.52**	0.61**	0.42**	0.30*
**Catalan**						
*N* = 113	Single word spelling	0.65**				
	Word reading	0.62**	0.80**			
	Reading comprehension	0.24*	0.44**	0.35**		
	Text productivity	0.48**	0.35**	0.35**	0.07	
	Text quality	0.66**	0.60**	0.60**	0.25*	0.52**

Stepwise multiple linear regression analyses were used to test models for predicting written text productivity and quality in the three languages. Independent variable were age, the transcription measures (handwriting fluency and spelling) and the reading measures (decoding and reading comprehension). Significant models emerged in all languages for text quality and in Catalan and English for productivity. Significant models and predictors can be found in Table 3. As the table shows, both productivity and text quality were predicted by age and transcription skills in both Catalan (only handwriting was a significant predctor) and English (both spelling and handwriting reached significance), and the largest variance was evident for the text quality measures (55 and 57%, respectively). By contrast for Spanish text quality is predicted by the two reading variables accounting for 42% of the variance but text productivity was not predicted by any of the measures in the current study.

**TABLE 3:** Final regression models reporting significant predictors written text measures for Catalan, English, and Spanish.

	Predictor	*B*	Std error	Beta	*t*	Sig	Model	Adjusted *R*^2^
**Productivity**								
Catalan	Handwriting fluency	0.54	0.22	0.29	2.09	–0.04	*F*(5,107) = 7.605, *p* < 0.001	0.23
English	Handwriting fluency	0.48	0.18	0.31	2.68	–0.009	*F*(l,85) = 7.1, *p* < 0.001	0.26
	Reading comprehension	1.02	0.38	0.39	2.64	0.009		
Spanish	No significant predictors						*F*(5,84) = 1.02, *ns*	0.001
**Text Quality**								
Catalan	Age	0.03	0.007	0.49	3.84	<0.001	*F*(5,107) = 27.82, *p* < 0.001	0.55
	Handwriting fluency	0.01	0.01	0.22	2.17	0.03		
English	Age	0.03	0.01	0.37	3.89	<0.001	*F*(5,85) = 23.87, *p* < 0.001	0.57
	Handwriting Fluency	–0.02	0.01	0.23	2.59	0.01		
	Spelling	0.04	0.02	0.31	2.19	0.001		
Spanish	Word Reading	0.01	0.003	0.49	2.98	0.004	*F*(5,84) = 13.12, *p* < 0.001	0.42
	Reading comprehension	0.05	0.02	0.23	2.48	0.02		

## Discussion

We examined patterns of spelling and narrative writing in three different age groups of Catalan, English, and Spanish speaking children. We predicted that the differences between the orthographies and morphological systems used by the children would impact on their relative spelling performance and, as a consequence, written productivity and writing quality. As predicted Spanish children were the better spellers on both the single word spelling assessment and in their written narratives. Indeed, the Spanish children produced very few spelling errors at any age, they were the most productive writers and wrote higher quality texts than the other two cohorts. The quality, but not the productivity, of their texts was predicted by their reading skills. By contrast, and as anticipated, the English children were challenged by spelling both at the single word level and in their written narratives. Overall their written text productivity was lower than those of the Catalan and Spanish children but the quality of the texts was similar to their Catalan peers. As predicted spelling skills were a significant constraint both in terms of productivity and quality. The spelling performance of the Catalan children was, as anticipated, not as good as the Spanish children but showed steady improvement across the three year groups. The difference between English and Catalan varied by type of spelling assessment. In contrast to single word spelling where English children were the most challenged group, in the narrative task the Catalan children produced both the highest number of misspelled words and the highest number of spelling errors overall in their written texts. This pattern remained consistent across all three year groups. Only handwriting predicted the productivity and quality of their written texts for the Catalan children.

In terms of quality and productivity, different developmental patterns were evident across the three languages. In Spanish, children were more productive at younger ages and this was reflected in the higher quality of their written texts. By contrast, both children writing in English and in Catalan demonstrated a more gradual pattern of development in terms of both productivity and quality, differences were evident between the two older year groups. Nonetheless, by the time children were in the last year of elementary school (Year 6) there were no differences between the cohorts in terms of the quality of their texts but the Catalan children were most productive.

We examined the factors which predicted productivity and quality for the three languages by using a series of multilinear regressions. As predicted reading was explained a significant proportion of the variance in the performance of Spanish children, while for English children spelling accounted for the most variance. The Spanish data, as has been demonstrated for other transparent orthographies (Babayigit and Stainthorp, 2011; Arfe et al., 2016) further highlight the need to consider wider factors that impact on the quality of children’s text. It is also important to note that once spelling and handwriting are fluent, at least for transparent orthographies, productivity no longer serves as a good discriminator of children’s writing skills.

In contrast to models of reading, models of writing have only recently begun to consider proximal and distal factors that impact on writing products (Dockrell, in press). Proximal factors, those directly related to the production of the written text (handwriting and spelling) have dominated our understanding of writing development in English; only small amounts of variance have been accounted for by more distal factors. The data from the Spanish sample clearly indicated that once challenges by proximal factors are overcome (as in transparent orthographies) distal factors are more important. The key role of these distal factors once transcription skills are in place warrants both further research and the refinement of current models of writing. Their importance also points to the potential role of distal factors in interventions to support writing development.

Why handwriting fluency affected both text productivity and text quality across all age groups in Catalan is not clear. Graphomotor execution is thought to be automatized around nine years of age (Afonso et al., 2017) but also susceptible to orthographic and morphological effects. Single word writing studies have shown that inter letter interval duration increases both at the inter syllable boundary (Alvarez et al., 2009) and in the presence of bound morphemes (Kandel et al., 2008). Lexical variables seem to have a more evident impact on handwriting when the sublexical route is used to spell and write words. Recently, a study examining word writing in Spanish found that orthographic consistency cascades into movement production in children. However, this effect was found only for children in Year 2 when writing was not been yet automated (Suarez-Coalla et al., 2018). However, the complexities of the Catalan language where the repertoire of multi-syllabic and multi-morphemic words interplay with a moderately opaque orthography may serve as a bottle-neck captured in handwriting fluency at this stage of development. Since the relationship between spelling and handwriting has been explored in word level studies, our findings indicate that these effects are evident at the text level as well, emphasizing the need to examine this interaction at the text production level and to explore if they are consistent across different languages and orthographies.

To further explore how different writing systems pose different challenges for developing writers, we examined the distribution of spelling errors that the children produced in their narrative writing. The Catalan children produced the highest number of misspelled words and the highest number of misspellings overall. A substantial number of the spelling errors they made, however, were accounted for the omission of an accent mark. The English children produced more root errors than the Spanish children and more affix errors than both Spanish and Catalan children. They were the only group that made more morphological errors than root errors. These results are consistent with previous findings in English by Bahr et al. (2012) that spelling affixed morphemes represents an increasingly difficult task as children progress through primary school.

The Catalan children also produced significantly more root errors but not affix errors than the Spanish children. This might indicate that the Catalan children relied on sublexical approach to spelling the word root but demonstrated the ability to analyze the morphological structure of the word as an alternative strategy to spell bound morphemes. Additionally, as anticipated, the Catalan children produced significantly more errors in the omission of accents than the Spanish children. The clear advantages in spelling experienced by the Spanish children further corroborate differences found in experimental studies that have demonstrated that shallow orthographic systems (e.g., comparison between Spanish and French) in addition to transparency at the phonemic level appear to support the storage of orthographic representations more easily (Carrillo et al., 2013). As such Spanish which has a clear syllabic structure and five vocalic phonemes facilitates phonemic awareness and the development of orthographic representations resulting in few root errors and affix errors and reducing the impact of spelling on text production. The finding that Catalan children produced as many root errors as the English children but fewer affix errors would support the view that learning to spell requires that the child perceives and integrates linguistic information onto orthographic representations and that there is an interaction between the characteristics of the language and the strategies children use for spelling. Here, the more salient morphology of Catalan supported a faster development of an accurate orthographic representation of the bound morphemes in this language. For instance, apparently, a child spelling the inflected form/pǝrsons/ →<per’sones> “persons-fem-pl” faces two equivalent difficulties of phoneme-grapheme inconsistency: /ǝ/ spelt <*e*>, both in the root and the affix. Similarly, a child spelling/ǝßε/ → <haver> “(auxiliar) to have” faces the challenge of representing a phonologically empty letter both in the root, for letter <h> and in the affix, for letter <r>. Both to solve the phoneme-grapheme inconsistency in the root of “persones” or to produce the phonologically empty <h> in the root of “haver,” the child will need to rely on lexical knowledge of these words. It appears, children in the early grades of primary school show capacity to analyse the word morphological, and the identification of the inflectional suffixes, here <-es>, expressing number, in <persones>, and <-er>, expressing the infinitive mode, in <haver>, provides the child with a helpful basis on which to produce the necessary spelling. These results are consistent with previous studies showing that the typological characteristic of the language affect the rate and path of development of an orthographic lexicon (Ravid and Gillis, 2006; Defior et al., 2008; Llaurado and Tolchinsky, 2016).

Our prediction that reading skills, which would arguably play a role in the development of an orthographic lexicon, would predict written text performance in Catalan was not supported by the present findings. One possible explanation is that the impact of the more distal factors cannot be seen until the transcription (handwriting) skills are consolidated. Free writing tasks provide children to choose the words in their text. As expected there was significant variability in their texts and therefore variation in the number of morphologically complex words used. This was particularly the case for derived words. Using a more controlled task such as a dictated text might provide a better way to explore this issue at this developmental point.

## Limitations

This study examines the contribution of transcription and literacy skills to the writing productivity and quality in three languages that differ both in the consistency of their orthographic systems and in the prominence of their morphologies. As with all studies which aim to examine cross-linguistic differences there are limitations. Firstly, we have only addressed differences in spelling performance according to the word morpheme (stem or affix) where they occur, a more fine grained analysis accounting for the type of spelling error would further inform the challenges posed by the characteristics of each writing system. Secondly, the text writing skills of children have been assessed by means of one single written text. There is increasing evidence that multiple tasks are a more valid indicator of writing proficiency than a single writing task. Thirdly, future studies should include a measure or a range of measures of oral language. Although existing studies typically report weak to moderate correlations between measures of oral language and the length and quality of children’s written products, the role of oral language in written text production should not be minimized as recent evidence suggests that oral sentence fluency supports written text generation over time and across languages (Savage et al., 2017). Fourthly, we depict development using cross-sectional data, longitudinal data are needed to fully establish the direction of the relationship between the different variables. Finally, although we statistically controlled for the small age differences between the Catalan and Spanish participants and English counterparts this is not ideal. However, this limitation is not specific to our sample, it reflects different school entry dates across systems and therefore difficult to overcome in the design of a comparative study. The limitations of the current study should inform future research.

## Conclusion

To conclude, this study is unique in its examination of the relationships of proximal and distal factors with two key measures for written text assessment: productivity and quality in three different languages and orthographies. Our findings demonstrate that the role of transcription and reading skills to developing writing is modulated by the characteristics of the writing system. As such findings established in studies conducted only in English cannot be generalized to other languages and orthographies. These data further support the need for more crosslinguistic studies to establish models of writing development that accurately depicts the process whereby children learn to use the written language flexibly for a wide range of communicative purposes. In addition to the theoretical implications of the findings, the results of this study also have implications for educational practice too. Writing continues to be the most common means by which children are assessed and the specific characteristics of the language and its orthography will impact on the reliability and validity of different approaches to assessment of the writing product.

## Data Availability Statement

The datasets generated for this study are available on request to the corresponding author.

## Ethics Statement

The studies involving human participants were reviewed and approved by Ethics Committee of Institute of Education. Written informed consent to participate in this study was provided by the participants’ legal guardian/next of kin.

## Author Contributions

AL organized the database. JD supervised the analysis. Both authors contributed to the design and conceptualization of the study, literature review, and discussion.

## Conflict of Interest

The authors declare that the research was conducted in the absence of any commercial or financial relationships that could be construed as a potential conflict of interest.
